# ^90^Y-PET/CT-based dosimetry after selective internal radiation therapy predicts outcome in patients with liver metastases from colorectal cancer

**DOI:** 10.1186/s13550-018-0419-z

**Published:** 2018-07-13

**Authors:** Hugo Levillain, Ivan Duran Derijckere, Gwennaëlle Marin, Thomas Guiot, Michaël Vouche, Nick Reynaert, Alain Hendlisz, Bruno Vanderlinden, Patrick Flamen

**Affiliations:** 1 Department of Nuclear Medicine, Jules Bordet Institute, Université Libre de Bruxelles, Rue Héger-Bordet 1, B-1000 Brussels, Belgium; 2 Department of Medical Physics, Jules Bordet Institute, Université Libre de Bruxelles, Rue Héger-Bordet 1, B-1000 Brussels, Belgium; 3 Department of Radiology, Hôpital St-Pierre, Jules Bordet Institute, Université Libre de Bruxelles, Rue Héger-Bordet 1, B-1000 Brussels, Belgium; 4 Department of Digestive Oncology, Jules Bordet Institute, Université Libre de Bruxelles, Rue Héger-Bordet 1, B-1000 Brussels, Belgium

**Keywords:** SIRT, ^90^Y-PET/CT, Chemorefractory mCRC, Dosimetry, Metabolic response, Survival

## Abstract

**Background:**

The aim of this work was to confirm that post-selective internal radiation therapy (SIRT) ^90^Y-PET/CT-based dosimetry correlates with lesion metabolic response and to determine its correlation with overall survival (OS) in liver-only metastases from colorectal cancer (mCRC) patients treated with SIRT. Twenty-four mCRC patients underwent pre/post-SIRT FDG-PET/CT and post-SIRT ^90^Y-PET/CT. Lesions delineated on pre/post-SIRT FDG-PET/CT were classified as non-metabolic responders (total lesion glycolysis (TLG)-decrease < 15%) and high-metabolic responders (TLG-decrease ≥ 50%). Lesion delineations were projected on the anatomically registered ^90^Y-PET/CT. Voxel-based 3D dosimetrywas performed on the ^90^Y-PET/CT and lesions’ mean absorbed dose (Dmean) was measured. The coefficient of correlation between Dmean and TLG-decrease was calculated. The ability of lesion Dmean to predict non-metabolic response and high-metabolic response was tested and two cutoff values (Dmean-under-treated and Dmean-well-treated) were determined using ROC analysis. Patients were dichotomised in the “treated” group (all the lesions received a Dmean > Dmean-under-treated) and in the “under-treated” group (at least one lesion received a Dmean < Dmean-under-treated). Kaplan-Meier product limit method was used to describe OS curves.

**Results:**

Fifty-seven evaluable mCRC lesions were included. The coefficient of correlation between Dmean and TLG-decrease was 0.82. Two lesion Dmean cutoffs of 39 Gy (sensitivity 80%, specificity 95%, predictive-positive-value 86% and negative-predictive-value 92%) and 60 Gy (sensitivity 70%, specificity 95%, predictive positive-value 96% and negative-predictive-value 63%) were defined to predict non-metabolic response and high-metabolic response respectively. Patients with all lesions Dmean> 39 Gy had a significantly longer OS (13 months) than patients with at least one lesion Dmean < 39 Gy (OS = 5 months) (p = 0.012;hazard-ratio, 2.6 (95% CI 0.98–7.00)).

**Conclusions:**

In chemorefractory mCRC patients treated with SIRT, lesion Dmean determined on post-SIRT ^90^Y-PET/CT correlates with metabolic response and higher lesion Dmean is associated with prolonged OS.

## Background

Selective internal radiation therapy (SIRT), also called radioembolization, based on intra-arterial embolization of yttrium-90 (^90^Y)-labelled microspheres is an established treatment of primitive or metastatic liver disease [1, 2]. The most common indications for SIRT are hepatocellular carcinoma, liver metastases from colorectal cancer (mCRC), intrahepatic cholangiocarcinoma and neuroendocrine tumours [1, 3].

The efficacy of SIRT depends on a preferential arterial vascularisation of liver tumours. Thus, in case of a good targeting of the lesions, SIRT delivers a high dose of radiation to the targeted volume sparing most normal liver parenchyma [1, 2, 4]. Over the past decade, SIRT in mCRC patients has been tested in at least 10 clinical trials as a salvage therapy or in combination with systemic chemotherapy. A beneficial effect on liver disease control was shown [1, 5].

The response to treatment is evaluated on metabolic and/or anatomic images. A minimum of 6 to 8 weeks after treatment is necessary to assess the metabolic response on FDG-PET/CT. For anatomical response evaluation, an even longer time scale is required to avoid wrong interpretation because of the intratumoural inflammation, haemorrhage and oedema peripheral to treated lesions [3]. Consequently, within this delay the disease can progress especially in case of aggressive diseases [3].

Recent preliminary data indicated that post-SIRT dosimetry correlated with FDG-PET-based metabolic response assessment performed 6–8 weeks after SIRT [6, 7]. The feasibility of ^90^Y imaging with PET in the hours following SIRT has recently been assessed as well as its quantitative performance [8–10]. Despite low statistics β + emission, ^90^Y-PET/CT enables the measurement for a patient-specific treatment dosimetry [11]. Therefore, post-SIRT dosimetry, if related to patient outcome, could become a valuable tool for early post-SIRT treatment adaptation.

The aim of this work was to confirm that post-SIRT ^90^Y-PET/CT-based dosimetry correlates with metabolic response and to determine its correlation with overall survival in liver-only mCRC patients treated with SIRT.

## Methods

### Study design and patient selection

This single-institution, retrospective trial enrolled patients with liver-only mCRC treated since 2013 with resin ^90^Y-microspheres with an assumed activity per sphere of 50–60 Bq (SIR-Spheres, Sirtex medical Ltd., Sydney, Australia). All included patients were progressive under FOLFOX and were scheduled for SIRT by the multidisciplinary tumour board. This trial was approved by the medical ethics committee.

### ^90^Y-microspheres therapy

Workup and treatment were performed following the current standard of practice and according to the manufacturer’s instructions [12–14]. However, the activity of ^90^Y-microspheres to administer was determined using the partition model instead of the standard body surface area (BSA) method [2–4, 15].

### FDG-PET/CT and ^90^Y-PET/CT imaging procedures

All patients were imaged in FDG-PET/CT EARL-accredited centres to guarantee image quality standardisation. Most of the FDG-PET/CT images were acquired using a General Electric (GE) Discovery 690 time-of-flight (TOF) PET system (kh_xar.30.V40_H_H64_G_GTLNI:9x6_lyso software). For five patients, baseline FDG-PET/CT images were acquired using a Siemens Biograph64-mCT (Syngo VG50A software) for four patients and a Siemens Biograph128-mCT (Syngo VG51C software) for one patient. Baseline and follow-up whole-body FDG-PET/CT were performed respectively before and 6–8 weeks after SIRT [3, 16]. Patients were required to have fasted for at least 6 h and to have blood glucose levels < 120 mg/dL (150 mg/dL for diabetic patients) before FDG injection. Images were acquired 60 min after injection (range 60–70 min and did not differ by more than 10 min from the uptake time for the baseline FDG-PET/CT) of 4.2 MBq/kg (range 3.6–4.8 MBq/kg).

^90^Y-PET/CT imaging was performed 21 h (range 20–23 h) after ^90^Y-microsphere administration. All ^90^Y-PET scans were acquired using a GE Discovery 690 TOF PET system (timing resolution of 544 ps), in 3D mode with an acquisition time of 1 h (30 min per bed position, two bed positions with an overlap of 13 mm) and a matrix of 192 × 192 pixels of 2.73 × 2.73 mm with a slice thickness of 3.27 mm. Images were reconstructed with the built-in GE VUE Point Fx algorithm, an ordered subset expectation maximisation algorithm with 18 iterations and 3 subsets, and were post-filtered with a 13.7-mm full width at half maximum Gaussian function. Attenuation, diffusion and resolution recovery (VPFX) corrections were applied according to the QUEST phantom study recommendations [8].

### FDG-PET/CT analysis

Metabolic response assessment was performed using dedicated commercial software (PET VCAR v.4.6; Advantage Workstation; GE Healthcare). Post-SIRT FDG-PET/CT was automatically registered to the pre-SIRT FDG-PET/CT.

#### Lesion delineation process

Lesions were delineated on baseline FDG-PET/CT using a threshold corresponding to twice the normal liver mean SUV at baseline measured in a 3-cm-diameter sphere located in the healthy liver parenchyma [16]. Bridging between two or more lesions was manually corrected. Residual disease was finally delineated using the same threshold. All delineated lesions were validated by an experienced nuclear medicine physician.

#### Criteria for identification of target lesions

The criteria were adapted from PERCIST. On baseline FDG-PET/CT, only lesions with the longest axial diameter > 2 cm were considered as target [17]. For baseline and follow-up FDG-PET/CT, lesions for which respiration artefacts were clearly visible (presence of lesion uptake within the lung parenchyma) were excluded from analysis.

#### Lesion-based metabolic response (mR) assessment

Total lesion glycolysis (TLG) and SUV_peak_ for each target lesion were measured on both FDG-PET/CT. The lesion-based mR to therapy was expressed as continuous variables representing the percentage decrease in both TLG (TLG-decrease) and SUV_peak_ (SUV_peak_-decrease) between baseline and follow-up FDG-PET/CT according to the following formulas: TLG-decrease (%) = ((TLG _Baseline_ − TLG _Follow-up_)/TLG _Baseline_) × 100 and SUV_peak_-decrease (%) = ((SUV_peak Baseline_ − SUV_peak Follow-up_)/SUV_peak Baseline_) × 100 [16, 18].

#### Lesion-based mR categorisation

Lesions were classified as non-mR (TLG-decrease < 15%) and high-mR (TLG-decrease ≥ 50%) [6, 16].

### Post-treatment absorbed dose analysis

Dosimetry was performed using dedicated dosimetry software (Planet Onco 3.0; Dosisoft®). The ^90^Y-PET/CT was anatomically registered to the pre-SIRT FDG-PET/CT using an automated rigid registration method. Registration was manually corrected in case of matching errors assessed by visual inspection.

#### Lesion dosimetry process

**Fig. 1:**
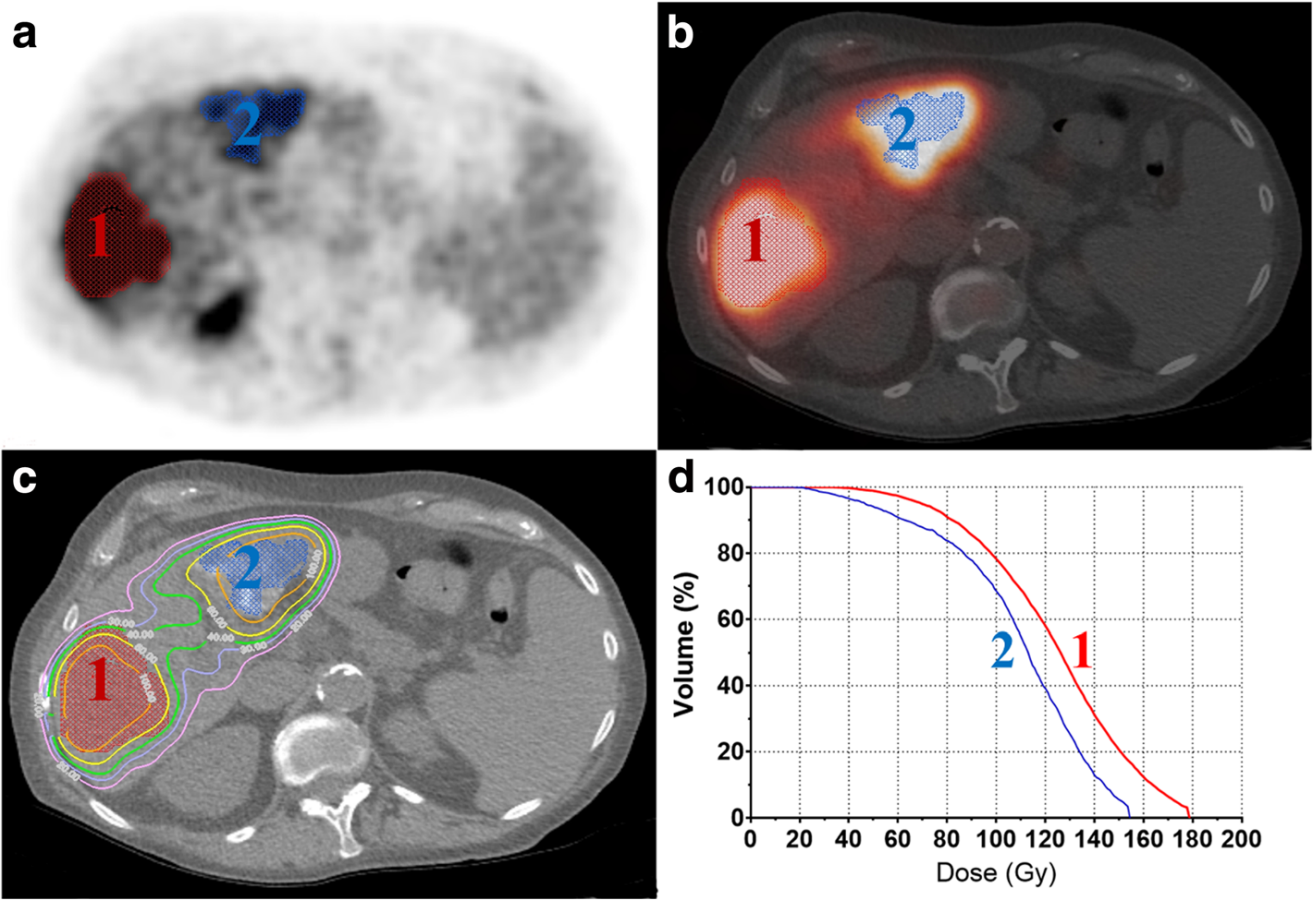
Post-treatment dosimetry process. **a** Baseline FDG-PET shows the delineated lesions (1 and 2). **b** Axial ^90^Y-PET/CT image on which two target lesion volumes have been projected, showing a high ^90^Y-microsphere uptake. **c** ^90^Y-microsphere-absorbed dose map showing that the lesions receive high absorbed doses (*D*_mean_-1 = 124 Gy and *D*_mean_-2 = 109 Gy). **d** Dose volume histograms of the two lesions (1 and 2)

Lesions delineated on baseline FDG-PET/CT were projected on the anatomically registered ^90^Y-PET/CT. Voxel-based time-integrated activity map (TIA-map) was computed by entering the injection date and time (image unit is in Bq/mL). Then voxel-based 3D dosimetry was performed on the ^90^Y–TIA-map using Voxel-S-value convolution [19, 20]. Finally, the dose volume histogram (DVH) was computed for each lesion and lesion mean absorbed doses (*D*_mean_) were determined. Figure 1 illustrates the post-treatment dosimetry process.

#### Post-treatment lesion *D*_mean_ cutoff value definition

Based on the two response cutoffs described above (high-mR and non-mR), post-treatment mean absorbed dose cutoffs for predicting the non-mR (*D*_mean_-under-treated) and the high-mR (*D*_mean_-well-treated) were determined using a ROC analysis.

#### Patient-based dichotomisation

We used the *D*_mean_-under-treated cutoff value to dichotomise the patient cohort into two groups. The “treated” group included patients with all the lesions receiving a *D*_mean_ superior to *D*_mean_-under-treated. The “under-treated” group included patients with at least one lesion receiving a *D*_mean_ inferior to *D*_mean_-under-treated.

### Statistical method

Descriptive analyses were performed to summarise baseline patient characteristics and ^90^Y-microsphere treatment. First quartile, median and third quartile were computed to describe lesion baseline TLG and SUV_peak_, follow-up TLG and SUV_peak_, TLG-decrease and SUV_peak_-decrease and finally *D*_mean_ distribution respectively. The metabolic response was first considered as a continuous variable. Scatter plots of TLG-decrease and SUV_peak_-decrease respectively with post-treatment *D*_mean_ were performed and a non-linear regression using a half maximal effective concentration EC50 dose-response model was then fitted to evaluate their relationship with the tumour *D*_mean_. In a second approach, the ability of lesions post-treatment *D*_mean_ to predict non-mR (TLG-decrease < 15%) and high-mR (TLG-decrease ≥ 50%) was tested and two cutoff values were determined using receiver operating characteristic (ROC) curves. *D*_mean_-under-treated and *D*_mean_-well-treated were defined for a specificity of 95% and highest sensitivity. Positive predictive value (PPV) and negative predictive value (NPV) were calculated for *D*_mean_-under-treated and *D*_mean_-well-treated. Finally the Kaplan-Meier product limit method was used to describe overall survival (OS) curves. OS was defined as the time between treatment and death or last contact (date of censoring). OS curves for the two patient groups described above (“treated” and “under-treated”) were computed. Hazard ratios were computed with the log-rank test and a two-sided *p* value < 0.05 was considered statistically significant. All statistical analyses were performed using the GraphPad 7.04 software (Prism®).

**Fig. 2:**
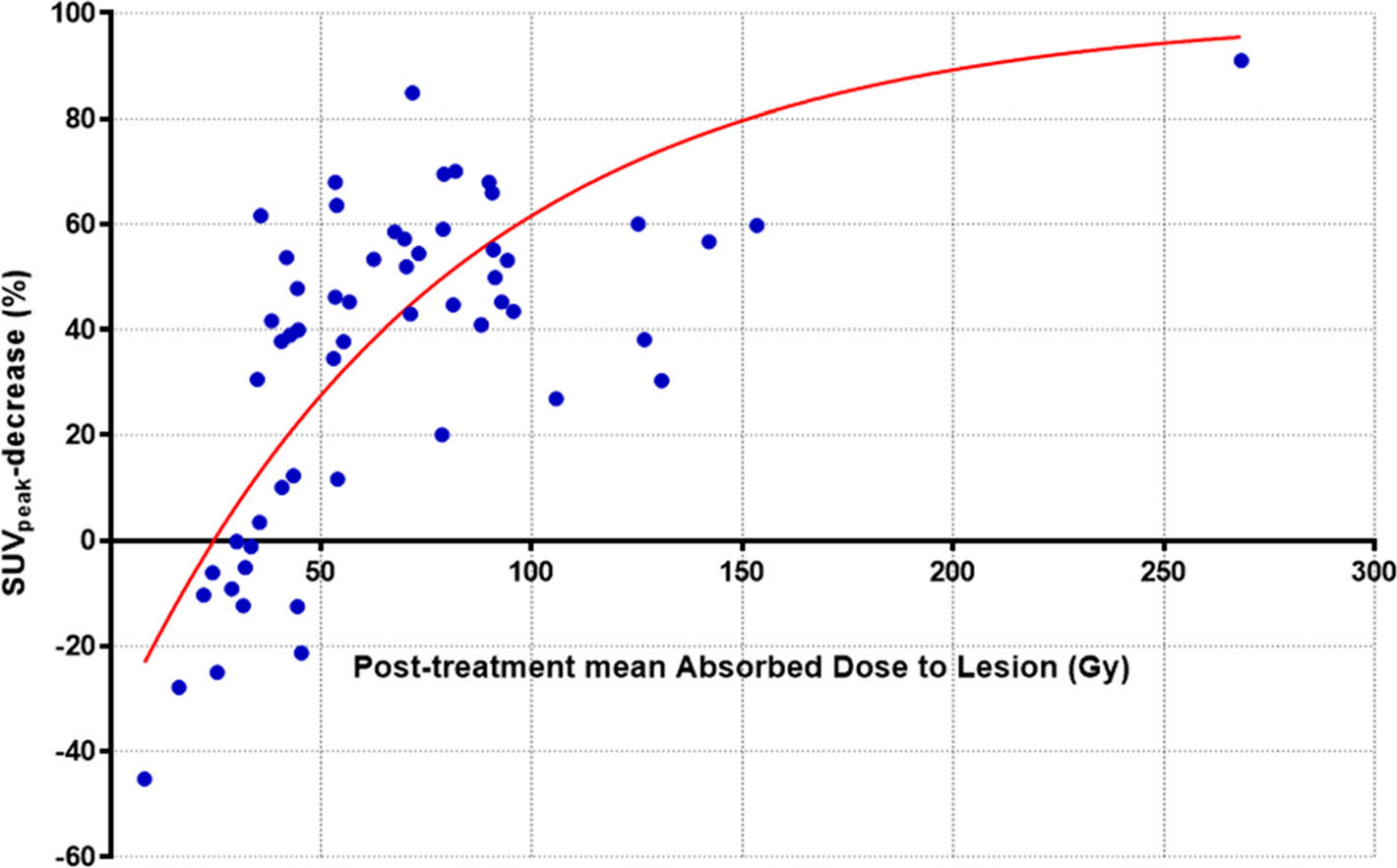
Regression analysis between post-treatment mean absorbed dose and the metabolic response assessed by SUV_peak_-decrease on a lesion by lesion basis (*R*^2^ = 0.52)

## Results

### Patients and treatment characteristics

Twenty-four patients progressive under FOLFOX were included in the study. Their baseline characteristics are presented in Table 1.

**Table 1:** Baseline characteristics of patients

Characteristic	*N* or mean ± SD
Sex	
Male	8 (33%)
Female	16 (67%)
Age (year)	66 ± 11
Previous systemic therapy	
Bevacizumab	14 (58%)
Cetuximab	3 (12%)
Irinotecan	5 (21%)
Panitunumab	1 (4%)
Tumour burden (% of whole liver)	
< 10%	16 (66%)
> 10%	8 (34%)
Metabolic tumour volume (ml)	99 ± 98
^90^Y-microsphere prescribed activity (MBq)	1120 ± 503
Treatment	
Whole liver	6 (25%)
Lobar	15 (63%)
Supra-selective	3 (12%)

### Lesion metabolic response and mean absorbed dose

A total of 134 lesions were delineated of which 77 lesions had to be excluded from analysis, because their diameter was inferior to 2 cm (75 lesions) or because their quantification was impaired by respiration artefacts (2 lesions). The characteristics of the 57 lesions eligible for analysis are presented in Table 2. With a median number of 3 lesions per patients (range 1 to 5), the median lesion post-treatment absorbed dose was 55 Gy (range 8 to 268 Gy). The median TLG-decrease was 96% (range − 1116 to 100%). The EC50 dose-response model yielded a coefficient of correlation *R*^2^ = 0.82 between the *D*_mean_ and TLG-decrease. The median SUV_peak_-decrease was 43% (range − 45 to 85%). The EC50 dose-response model yielded a coefficient of correlation *R*^2^ = 0.52 between the *D*_mean_ and SUV_peak_-decrease. Figure 2 shows the regression analysis between post-treatment mean absorbed dose and the SUV_peak_-decrease.

**Table 2:** Characteristics of lesions

	Min	Q1	Median	Q3	Max
Baseline TLG (g)	10	36	71	165	1228
Baseline SUVpeak (g/ml)	3	5	6	8	13
Follow-up TLG (g)	0	0	7	61	5115
Follow-up SUVpeak (g/ml)	1	3	4	5	14
TLG-decrease (%)	− 1116	8	96	100	100
SUVpeak-decrease (%)	− 45	11	43	57	91
Lesion mean absorbed dose (Gy)	8	39	55	89	268

**Fig. 3:**
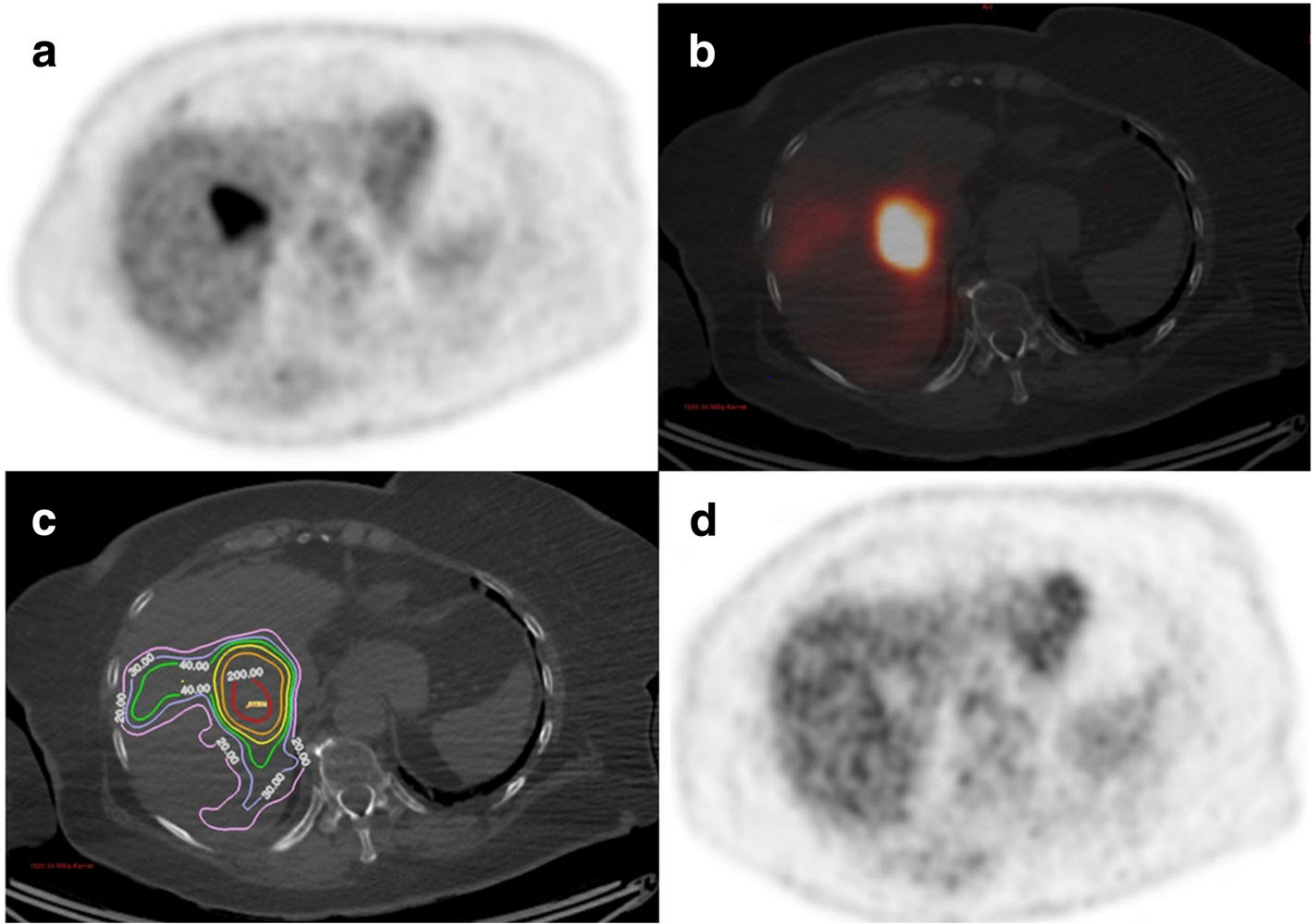
Registered axial images of a patient with liver mCRC. **a** Baseline FDG-PET shows the target lesion located in segment VIII. **b** ^90^Y-PET/CT images after supra-selective SIRT administered in the right hepatic artery, showing a high ^90^Y-microsphere uptake. **c** ^90^Y-microsphere absorbed dose map showing that the lesion receives a high absorbed dose (*D*_mean_ = 268 Gy). **d** Follow-up FDG-PET obtained at 6 weeks post-SIRT indicating a complete response

### Prediction of the metabolic response

The results of the univariate analysis for predicting a TLG-decrease of less than 15% are presented in Table 3. Based on ROC curve analysis and for a specificity of 95% (95% CI 82.84–99.42), we defined the lesion *D*_mean_-under-treated = 39 Gy in order to predict the non-mR. The associated following results were found: sensitivity 80% (95% CI 51.91–95.67), PPV 86% and NPV 92%.

**Table 3:** Results of the univariate analysis for predicting a TLG-decrease of less than 15%

		TLG-decrease ≤ 15	TLG-decrease > 15	Area under the ROC curve	*p* value
Lesion *D*_mean_ (Gy)	*N*	15	42	0.97	< 0.0001
	Mean ± SD	31 ± 11	80 ± 42		
	Min–max	8–53	36–268		

The results of the univariate analysis for predicting a TLG-decrease of more than 50% are presented in Table 4. Based on ROC curve analysis and for a specificity of 95% (95% CI 75.13–99.87), we defined the lesion *D*_mean_-well-treated = 60 Gy in order to predict the high-mR. The associated following results were found: sensitivity 70% (95% CI 53.02–84.13), PPV 96% and NPV 63%. Figure 3 illustrates the metabolic response of a lesion receiving a *D*_mean_ superior to 60 Gy.

**Table 4:** Results of the univariate analysis for predicting a TLG-decrease of more than 50%

		TLG-decrease ≤ 50	TLG-decrease > 50	Area under the ROC curve	*p* value
Lesion *D*_mean_ (Gy)	*N*	20	37	0.92	< 0.0001
	Mean ± SD	37 ± 16	84 ± 43		
	Min–max	8–78	36–268		

**Fig. 4:**
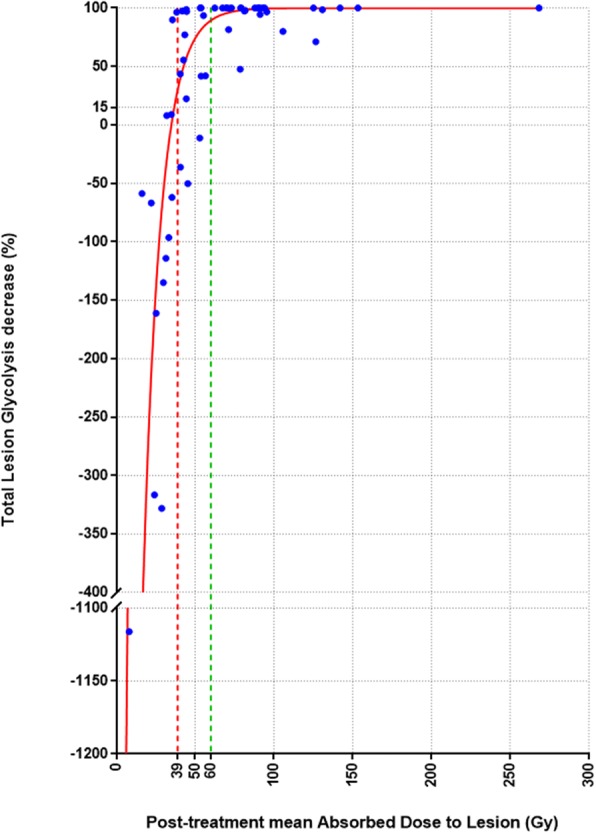
Regression analysis between post-treatment mean absorbed dose and the metabolic response assessed by TLG-decrease on a lesion by lesion basis (*R*^2^ = 0.82). Red and green lines represents post-treatment mean absorbed dose cutoff values for predicting respectively non-mR (39 Gy) and high-mR (60 Gy)

Figure 4 shows the regression analysis between post-treatment mean absorbed dose and the TLG-decrease with the defined dose cutoff.

### Analysis of overall survival curves

#### Global overall survival

The overall survival of our cohort included the 24 patients. The median OS was 9 months. Among the 24 patients, 4 were censored because they were lost to follow-up.

#### Lesion mean absorbed dose: a predictor of overall survival

**Fig. 5:**
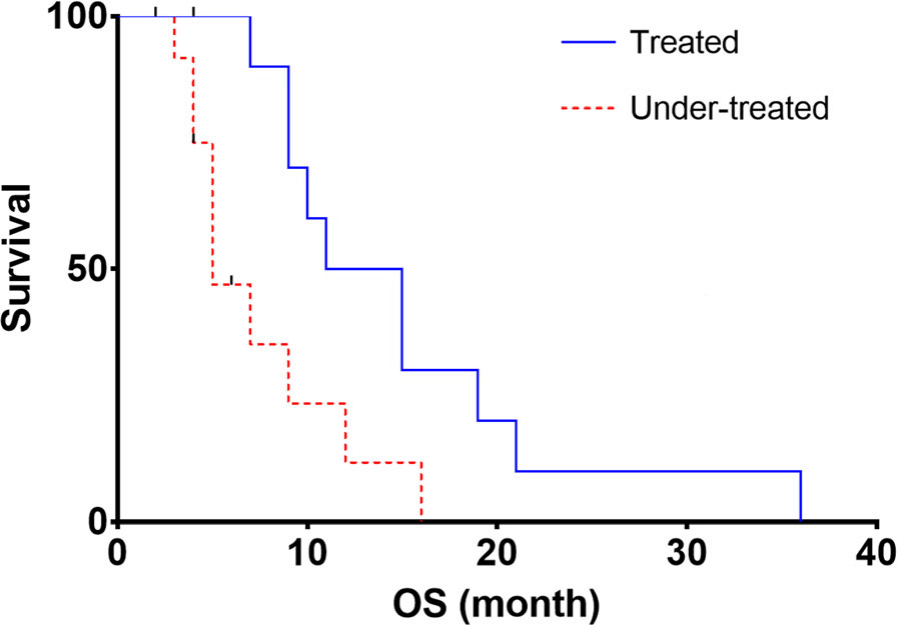
OS curves estimated by the Kaplan-Meier method according to the treated group (all the lesions received a mean absorbed dose superior to 39 Gy) versus the under-treated group (at least one lesion received a mean absorbed dose inferior to 39 Gy)

OS curves revealed a significant difference (*p* = 0.012) between median OS of treated patients (*N* = 11) and under-treated patients (*N* = 13), 13 versus 5 months and a hazard ratio (HR) of 2.6 (95% CI 0.98–7.00). Figure 5 shows the OS curves.

## Discussion

The aim of this work was to confirm that post-SIRT ^90^Y-PET/CT-based dosimetry correlates with metabolic response and to determine its correlation with overall survival in liver-only mCRC patients treated with SIRT.

Our results confirmed that there is a correlation between the lesion post-treatment *D*_mean_ and the metabolic response assessed by TLG-decrease (*R*^2^ = 0.82). Two tumour mean absorbed dose cutoffs of 39 and 60 Gy were defined for predicting respectively the non-metabolic response (less than 15% TLG-decrease) and a high metabolic response (more than 50% TLG-decrease). Our results also demonstrated that patients in which all the lesions had a *D*_mean_ superior to 39 Gy had a significant prolonged OS compared to the patients in which at least one lesion had a *D*_mean_ inferior to 39 Gy, 13 vs 5 months respectively.

Using the partition model for prescribing the activity to administer, we obtained higher tumour absorbed doses compared to what has been reported in similar studies that are based on BSA method [6, 7]. We found that 50% of the lesions had a post-treatment mean absorbed dose superior to 55 Gy and only 25% inferior to 39 Gy. These mean absorbed dose values were assessed for a given ^90^Y-microsphere specific activity, which could differ from one centre to the other, e.g. between centres using resin or glass ^90^Y-microspheres [21]. Also, to obtain the equivalent values in another radiotherapy technique, such as external beam radiation therapy (EBRT), the biological effective dose (BED) for the liver must be used to convert ^90^Y-microsphere absorbed dose values [22]. A significant metabolic response (TLG-decrease > 50%) was achieved in 65% of the lesions and half of the metastases showed a metabolic response superior to 96% TLG-decrease even though patients in our trial were heavily pre-treated. In their prospective study, van den Hoven et al. showed, in a cohort similar to ours, that metabolic response was achieved in less than half of metastases when using the BSA method [6]. They emphasised the need of a more personalised approach to optimise tumour dose delivery in mCRC patients. Our study demonstrated that using the partition model to prescribe the activity to administer was clinically feasible and could lead to a higher lesion metabolic response rate compared to what has been reported in the study of van den Hoven et al. The entire procedure (partition model dosimetry, post-SIRT dosimetry and multidisciplinary meetings between nuclear medicine physicians and physicists, and interventional radiologists) requested an average of 4 h of work per patient, which was compatible with our clinical routine.

In several studies, TLG-decrease is used for evaluating the metabolic response to therapy and is used, as endpoint, as surrogate of common clinical endpoint such as OS or progression-free survival (PFS) in mCRC patients [6, 7, 16, 23, 24].TLG characterises the mass of active tumour cells in the lesion and their aggressiveness [25]. Thus, TLG-decrease represents the change in tumour aggressiveness and tumour mass due to cancer cell death during treatment. In their study, Lim et al. found that TLG measurement can predict treatment outcome of regorafenib in mCRC. They reported that baseline TLG can be used as a sensitive prognostic metabolic biomarker and that a greater TLG-decrease is associated with both better PFS and better OS [24].This relationship could be also verified in SIRT of liver metastases as suggested in exploratory survival analysis of van den Hoven et al. [6]. Despite the use of different methods for measuring TLG-decrease and lesion *D*_mean_, our correlation between lesion ^90^Y-PET/CT-based *D*_mean_ and TLG-decrease confirms the findings of van den Hoven et al. and Willowson et al. Nevertheless, a pitfall when using the TLG-decrease for evaluating the metabolic response is that it is highly sensitive to the lesion delineation process. We noticed that some lesions were still visible at follow-up but they were not segmented because their SUV were under the delineation threshold. A TLG-decrease of 100% was wrongly attributed to these lesions. However, we also obtain a good correlation between SUV_peak_-decrease and *D*_mean,_ in which our delineation process has no influence. Therefore, when SUV in residual disease is under the delineation threshold, the SUV_peak_ at follow-up could be used to compute TLG-decrease.

Based on TLG-decrease, two post-treatment tumour *D*_mean_ cutoff values were defined at 39 and 60 Gy for predicting non-metabolic response (15% TLG-decrease) and high metabolic response (50% TLG-decrease) respectively. It might be of clinical interest to classify lesions into non-treated, moderately treated and well-treated categories depending on lesion *D*_mean_. The intermediate category between 39 and 60 Gy, where lesion metabolic response is more variable, suggests that dose effects on tumour cells are not fully deterministic in this range and that other parameters should be taken into account. Depending on their phenotype and their microenvironment, lesions could be either more sensitive or more resistive to ^90^Y irradiation, from one patient to another or even from one lesion to another for a given patient. In their study, Walrand et al. showed that haemoglobin level measured on the day of SIRT procedure has an impact on the early tumour response in different types of liver metastases [26]. There are many other potential prognostic or predictive factors for tumour radiosensitivity or radioresistance that may have significant impact on tumour response to SIRT [7]. A better understanding of the underlying radiobiological mechanisms is needed. The heterogeneity of absorbed dose inside the lesion could also be an important factor in predicting metabolic response of lesion receiving a *D*_mean_ between 39 and 60 Gy. For example, the minimal absorbed dose inside a lesion could be an interesting parameter to investigate. The *D*_mean_ cutoff values defined in this work allow for implementing early treatment adaptation in case of under or moderate treatment of the lesion. If the lesion received less than 39 Gy, a salvage treatment strategy should be realised. Complementary SIRT treatment could be evaluated by reassessing the angio-CT to see whether no other arterial approach is feasible. In moderately treated lesion, between 39 and 60 Gy, a boost treatment could be performed to tilt the balance in favour of a significant response, e.g. using EBRT or immunotherapy.

The correlation of post-SIRT dosimetry with OS has seldom been studied. In their study, van den Hoven et al. found that patients with a liver tumour dose exceeding 60 Gy had a longer median OS than those with lower liver tumour dose [6]. However, average liver tumour dose estimation does not reflect the inter-lesion dose heterogeneity.

In state-of-the-art methods, the lesion with the worst prognostic (highest SUV, dimensions) is the most influential in the patient OS and determines the patient response to treatment [17, 27, 28]. On this basis, we hypothesised that the lesion receiving the lowest mean absorbed dose was the most influential in the patient OS. Therefore, as non-metabolic response lesions (TLG-decrease < 15%) might be the ones responsible for the patient’s death, we used the cutoff value of 39 Gy defined to predict the non-metabolic response. Recently, a large randomised multicentre trial by Wasan et al. showed a non-benefit of the combination of SIRT with FOLFOX in terms of PFS and OS even though better liver-specific disease control and better radiological response were demonstrated [5]. The prescribed ^90^Y-microsphere activity to administer was computed using the standard BSA method corrected with the percentage of tumour involvement and the magnitude of liver-to-lung shunting. Determining the optimal activity to administer is crucial for SIRT effectiveness as we demonstrated that there is a relationship between lesion mean absorbed dose and both metabolic response and OS. In their retrospective analyses, Grosser et al. demonstrated that patients’ BSA and liver volume showed only a moderate correlation and that tumour burden percentage contributed little to the prescribed activity [29]. The authors emphasised that BSA method results in a significantly lower computed ^90^Y-microsphere activity to administer which may potentially result in underdosage in patients with a larger liver. For these reasons, the absence of benefit in terms of PFS and OS for mCRC patients treated with SIRT and FOLFOX could be explained, in part, by underdosages engendered by the use of BSA method. Therefore, ^90^Y-microsphere activity to administer should be determined with a personalised framework taking into account the patient-specific therapeutic window in order to maximise the absorbed dose to the lesions while minimising the absorbed dose to the organs at risk.

## Limitations of this study

Limitation of the study is that it is a retrospective study from a single centre. It is uncertain whether the results would be reproduced without the use of a personalised pre-SIRT dosimetry for prescribing the activity of ^90^Y-microspheres to administer. Also, the small number of patients (*n* = 24) and analysed lesions (*n* = 57) limits the application of our results in an external dataset. Prospective validation of our results must be performed in multicentre studies. Finally, this study is focussed on liver-only mCRC patients treated with resin 90Y-microspheres; similar studies should be performed in other liver diseases and using other treatment devices.

## Conclusions

In chemorefractory mCRC patients treated with SIRT, absorbed dose determined on post-SIRT ^90^Y-PET/CT correlates with metabolic response, and higher lesion mean absorbed doses are associated with prolonged OS. These relationships underline the need for a personalised process in order to optimise the activity of ^90^Y-microspheres to administer. Post-SIRT ^90^Y-PET/CT-based dosimetry enables rapid and precise prediction of SIRT efficacy within the 24-h post-treatment, allowing early treatment adaptation in case of undertreatment of the lesions.
